# Epstein-Barr virus DNA seropositivity links distinct tumoral heterogeneity and immune landscape in nasopharyngeal carcinoma

**DOI:** 10.3389/fimmu.2023.1124066

**Published:** 2023-02-13

**Authors:** Wangzhong Li, Shuhui Lv, Guoying Liu, Nian Lu, Yaofei Jiang, Hu Liang, Weixiong Xia, Yanqun Xiang, Changqing Xie, Jianxing He

**Affiliations:** ^1^ Department of Thoracic Surgery and Oncology, The First Affiliated Hospital of Guangzhou Medical University, State Key Laboratory of Respiratory Disease, National Clinical Research Center for Respiratory Disease, Guangzhou Institute of Respiratory Health, Guangzhou, China; ^2^ Department of Nasopharyngeal Carcinoma, Sun Yat-sen University Cancer Center, The State Key Laboratory of Oncology in South China, Collaborative Innovation Center for Cancer Medicine, Guangdong Key Laboratory of Nasopharyngeal Carcinoma Diagnosis and Therapy, Guangzhou, China; ^3^ Department of Ultrasound, The Fifth Affiliated Hospital of Sun Yat-sen University, Zhuhai, China; ^4^ Department of Radiation Oncology, Sun Yat-sen Memorial Hospital, Guangzhou, China; ^5^ Thoracic and GI Malignancies Branch, Center for Cancer Research, National Cancer Institute, National Institutes of Health, Bethesda, MD, United States

**Keywords:** nasopharyngeal carcinoma, Epstein-Barr virus DNA, single-cell RNA sequencing, tumor microenvironment, tumoral heterogeneity, immune landscape

## Abstract

**Background:**

Epstein-Barr virus (EBV) DNA seronegative (Sero-) and seropositive (Sero+) nasopharyngeal carcinoma (NPC) are distinctly different disease subtypes. Patients with higher baseline EBV DNA titers seem to benefit less from anti-PD1 immunotherapy, but underlying mechanisms remain unclear. Tumor microenvironment (TME) characteristics could be the important factor affecting the efficacy of immunotherapy. Here, we illuminated the distinct multicellular ecosystems of EBV DNA Sero- and Sero+ NPCs from cellular compositional and functional perspectives at single-cell resolution.

**Method:**

We performed single-cell RNA sequencing analyses of 28,423 cells from ten NPC samples and one non-tumor nasopharyngeal tissue. The markers, function, and dynamics of related cells were analyzed.

**Results:**

We found that tumor cells from EBV DNA Sero+ samples exhibit low-differentiation potential, stronger stemness signature, and upregulated signaling pathways associated with cancer hallmarks than that of EBV DNA Sero- samples. Transcriptional heterogeneity and dynamics in T cells were associated with EBV DNA seropositivity status, indicating different immunoinhibitory mechanisms employed by malignant cells depending on EBV DNA seropositivity status. The low expression of classical immune checkpoints, early-triggered cytotoxic T-lymphocyte response, global activation of IFN-mediated signatures, and enhanced cell-cell interplays cooperatively tend to form a specific immune context in EBV DNA Sero+ NPC.

**Conclusions:**

Collectively, we illuminated the distinct multicellular ecosystems of EBV DNA Sero- and Sero+ NPCs from single-cell perspective. Our study provides insights into the altered tumor microenvironment of NPC associated with EBV DNA seropositivity, which will help direct the development of rational immunotherapy strategies.

## 1 Introduction

Nasopharyngeal carcinoma (NPC) is a type of tumor derived from epithelial cells of the nasopharynx. Epstein-Barr virus (EBV) infection is the predominant pathogenic factor of NPC (1). Integrated EBV DNA can be detected in almost all EBV-associated NPC (2). The EBV DNA fragments released from infected host cells during replication or apoptosis offer rationales for EBV DNA detection using serum samples in managing NPC (3–5). Our previous studies have indicated that circulating EBV DNA load is a valuable predictive biomarker for treatment response and outcomes (6–8). EBV DNA seronegative (Sero-) and seropositive (Sero+) NPCs might represent two distinct subtypes of the disease (8). However, the intrinsic feature of NPC that connects with EBV DNA seropositivity has not been illuminated.

Recently, several clinical trials have demonstrated impressive antitumor effects of immune checkpoint inhibitors (ICIs) in patients with advanced NPC (9). However, the objective response rate of immunotherapy is suboptimal and only about 20-30%. *Post-hoc* analysis of the POLARIS-02 study showed that patients with higher baseline EBV DNA titers benefit less from anti-PD1 immunotherapy, but the underlying mechanisms remain unclear (10, 11). This indicates there is a unique tumor microenvironment (TME) in NPC with detectable EBV DNA in comparison to the one with undetectable EBV DNA. A better understanding of the TME heterogeneity between EBV DNA Sero- and Sero+ NPCs would be critical for the rational development of optimal therapeutic strategies in the future.

Single-cell RNA sequencing (scRNA-seq) can dissect transcriptional features at single-cell resolution to reflect cellular heterogeneity (12, 13). Several studies have used scRNA-seq to depict the TME in NPC, demonstrating a complex landscape involved in intratumoral heterogeneity, immune dynamics, and cell-cell interplays (14–17). However, these studies mainly compared the TME between malignant and nonmalignant samples. No analysis is available to illuminate the atlas of the TME in NPC with different EBV DNA statuses. Here, for the first time, we comprehensively compared the multicellular ecosystem of NPC with different EBV DNA seropositivity statuses. Our data provided in-depth insights into the altered TME of NPC associated with EBV DNA seropositivity, which will help develop rational immunotherapy strategies.

## 2 Materials and methods

### 2.1 Patient samples

The analyzed scRNA-seq data were deposited at the Gene Expression Omnibus database under accession code: GSE150430 (14). All samples were collected at Sun Yat-sen University Cancer Center. Written informed consent was obtained from all participants. Ethical approval was obtained from the Institutional Review Board of Sun Yat-sen University Cancer Center. The inclusion criteria for NPC samples we used were (1):. pathologically confirmed NPC (2); treatment-naive (3); EBV positive as confirmed using *in situ* hybridization of EBV encoded small RNAs in tumor tissue (4); available baseline EBV DNA data. Ten EBV-related NPC samples and one chronic nasopharyngitis sample were included in the final analysis. The study samples’ sequencing parameters and patient characteristics are summarized in **Table S1** and **Table S2**, respectively. EBV DNA Sero- was defined as undetectable EBV DNA in peripheral blood. EBV DNA Sero+ was defined as detectable EBV DNA in plasma with a titer of 10^3^ copies/mL or greater because of the detection limits of the plasma EBV DNA assays used in the study.

### 2.2 ScRNA-seq data processing

The preprocessed gene expression matrices for study samples were converted to a Seurat object using the Seurat package (version 4.1.0) in R (18). Low-quality cells with unique molecular identifiers < 200, expressed genes > 9000 or < 200, or mitochondrial genes > 20%, were removed. In the remaining cells, the gene expression dataset was normalized using the NormalizeData function and scaled with linear regression using the ScaleData function. In data scaling, the variables used to regress were “S.Score” and “G2M.Score” calculated by the CellCycleScoring function (19). The included features were the top 6000 highly variable genes selected by the FindVariableFeatures function.

### 2.3 Determination of major cell types and their subpopulations

The selected HVGs were summarized by principle component analysis (PCA) to reduce dimensionality, and the first 20 principle components were further projected using the Uniform Manifold Approximation and Projection (UMAP) (20). The number of principal components used was determined by an Elbow plot. We performed cell clustering using the FindClusters function with default parameters. The cell clusters were annotated as different major cell types based on DEGs and well-recognized marker genes. For sub-clustering analysis, we performed the second-round PCA reduction and UMAP projection separately on cells within each major cell type. The number of principal components and resolution used in each major subtype were dataset-dependent. The second-round dimension reduction and clustering revealed 28 distinct subpopulations in T/NK, B, and myeloid cells.

### 2.4 Identification of DEGs and gene set enrichment analysis

DEGs for each cluster were identified with the FindAllMarkers function using the MAST test. The differentially over-expressed genes in the specific group compared to the other group were identified with the FindMarkers function using the MAST test. We used the clusterProfiler package (version 4.0) for GO terms and KEGG pathways enrichment analysis. We also applied the Gene Set Variation Analysis (GSVA) implemented in the GSVA package (version 1.40.1) to estimate the pathway activities. Differences in pathway activities between groups were calculated using a linear model provided with the Limma package (version 3.48.3).

### 2.5 Quantification of differences in transcriptional signatures between major cell types in EBV DNA Sero- and Sero+ NPCs

We assessed differences in transcriptional profiles across major cell types in EBV DNA Sero- and Sero+ NPCs using the Bhattacharyya distance method implemented in the distdimscr package (version 0.0.0.0.9) (21, 22). The distdimscr quantifies these differences by examining the distance between cell types in a high-dimensional space. Here, we only measured distances between cell types that had 300 or more cells present across all samples from each tumor type (here, EBV DNA Sero- *vs*. Sero+) in PCA. We embedded all cell types in a new PCA space based on highly variable genes and retained the top 10 PCs for subsequent analysis. We then sampled 300 cells from each tumor type for 100 replicates. The Bhattacharyya distance between cells sampled regardless of EBV DNA status was used to generate a background distribution for statistical comparison. The Wilcoxon rank-sum test was conducted to compare the EBV DNA Sero- *vs*. Sero+ cell types against the random selection for each cell type.

### 2.6 Single-cell copy-number variation and clonality analysis

The large-scale chromosome CNVs in epithelial cells were inferred from raw gene expression matrices using the inferCNV package (version 1.10.1) (19). We performed inferCNV analysis with the following parameters: “denoise”, default Hidden Markov Model (HMM) settings, “subclusters” mode with “random_trees” method, and a value of 0.1 for “cutoff”. Epithelial cells extracted from the normal sample were used as reference data. To distinguish malignant and non-malignant epithelial cells in NPC samples, K-means clustering analysis was performed separately on epithelial cells derived from each of the eight tumor samples and the non-tumor sample according to CNV scores. Cells of the tumor samples in the cluster that predominantly contained cells from the normal sample were regarded as non-malignant cells. In contrast, the cells with high CNV scores in other clusters were identified as malignant cells. Consulting with the genome annotation data (GRCh38 version), each CNV was further converted into a p- or q-arm level change according to its position and annotated as either a gain or a loss. After data conversion, subclones containing identical arm-level CNVs were collapsed, and trees were restructured to represent subclonal CNV architecture. The UPhyloplot2 plotting algorithm was used for the visualization of intra-tumor evolutionary trees (23, 24).

### 2.7 Intratumoral transcriptional heterogeneity and intertumor co-expression modules

Consensus Non-negative Matrix factorization (cNMF, version 1.4) implemented by Python was used to infer gene expression programs from the scRNA-Seq data (25). A total of 50 gene expression programs from the eight tumor samples with at least 50 epithelial cells. We next determine the underlying biological functions of each of the 50 gene expression programs using Gene Set Enrichment Analysis (GSEA) based on their top 50 rank genes. Hierarchical clustering based on pairwise correlation coefficients calculated from each possible GEP pair was further performed to identify potential co-expression modules. We identified six potential co-expressed gene modules. To determine the gene signature of each co-expression module, we combined the top 50 genes of each meta-gene expression program. We calculated the average weighted score of each gene according to the NMF load. Finally, the top 50 genes with the highest weighted scores were defined as the gene signature of the corresponding co-expression module.

### 2.8 Calculation of enrichment scores for gene signatures or modules

For scRNA-seq data, we calculated the module/signature enrichment scores on a single-cell level using the AddModuleScore function provided by the Seurat package. For bulk RNA-seq data, we calculated module/signature enrichment scores for each sample using the Mann-Whitney U statistic provided by the UCell package (version 1.44.2). Details on gene signature datasets we used were summarized in the supplement (**Table S3**). These gene data sets were collated based on reviewing relevant literature reports.

### 2.9 Correlation of malignant signatures and survival

We assessed the associations of the malignant signatures and survival outcomes in a bulk RNA-seq cohort (GSE102349), which consisted of 113 NPC samples (26). Among 113 NPC samples, 88 samples with available survival data were used for subsequent survival analysis. We determined the optimal cutpoints for gene expression signatures using the maximally selected rank statistics from the ‘maxstat’ R package (version 0.7.25).

### 2.10 Single-cell regulatory network inference and clustering analysis

We inferred gene regulatory networks using the SCENIC package (version 1.2.4) implemented in R (27). The gene regulatory networks are inferred based on co-expression modules and TFs motif enrichment analysis from scRNA-seq data. We calculated and ranked the activities of TFs, as measured by Regulon Specificity Score (RSS), using the AUCell package (version 3.12) (28).

### 2.11 Trajectory analysis

The pseudotime trajectories of malignant cells and CD8^+^ T cells were inferred with the Monocle 2 package (version 2.22.0). Only genes with high dispersion across cells were used in the trajectory analysis. Dimension reduction was performed with the reduceDimension function using the “DDRTree” method and a value of 2 for “max_components”. We ordered and visualized cells using the plot_cell_trajectory function. Genes that separated cells into two differentiated cell states were identified by the Branched Expression Analysis Modeling (BEAM) implemented by the BEAM function. Branch-dependent genes with a q-value less than 10^-4^ were classified with hierarchical clustering using the plot_genes_branched_heatmap function. Genes that changed along with the pseudotime were identified with the differentialGeneTest functiom and visualized with plot_pseudotime_heatmap function. The GO terms enrichment analysis for the genes in each cluster was performed to characterize the biological functions.

The CytoTRACE (Cellular (Cyto) Trajectory Reconstruction Analysis using gene Counts and Expression) computational method, another robust trajectory analysis tool provided in the CytoTRACE package (version 0.3.3), was performed to validate the Monocle 2 inferred linear transition (29). CytoTRACE can infer the direction of differentiation from scRNA-seq data without any prior knowledge.

### 2.12 RNA expression-based stemness index of malignant cells

We assessed the stemness of malignant cells using the RNA expression-based stemness index (mRNAsi). The mRNAsi was proposed by Malta et al. using the One Class Linear Regression (OCLR) algorithm based on pluripotent stem cell samples from the Progenitor Cell Biology Consortium dataset (https://www.synapse.org) (30). The generated mRNAsi is a standardized value between 0 and 1; the closer it is to 1, the stronger the cell’s stemness. We applied the model to the scRNA-seq dataset to calculate the mRNAsi of each malignant cell.

### 2.13 Cell-cell communication

We performed ligand-receptor analyses using the CellChat package (version 1.1.3) to compute cell-cell communication from single-cell transcriptomics data. CellChat has built ligand-receptor interactions for human in the CellChat database. The database considers known interactions between ligands, receptors, and cofactors. CellChat quantifies the cell-cell communication probability and identifies significant cell-cell interactions. CellChat can compare cell-cell communication patterns between two scRNA-seq datasets and identify significant signaling changes across different conditions.

### 2.14 Statistics analysis

All statistical analyses were performed using R (version 4.1.0) or Python (version 3.9.5). The differences between groups were analyzed by chi-square test, student t test, Wilcoxon test, Pearson correlation test, and Kruskal-Wallis test, when appropriate. All tests were two-sided and P < 0.05 was considered as statistical significance.

## 3 Results

### 3.1 Single-cell expression atlas in EBV-associated NPC

**Figure 1:**
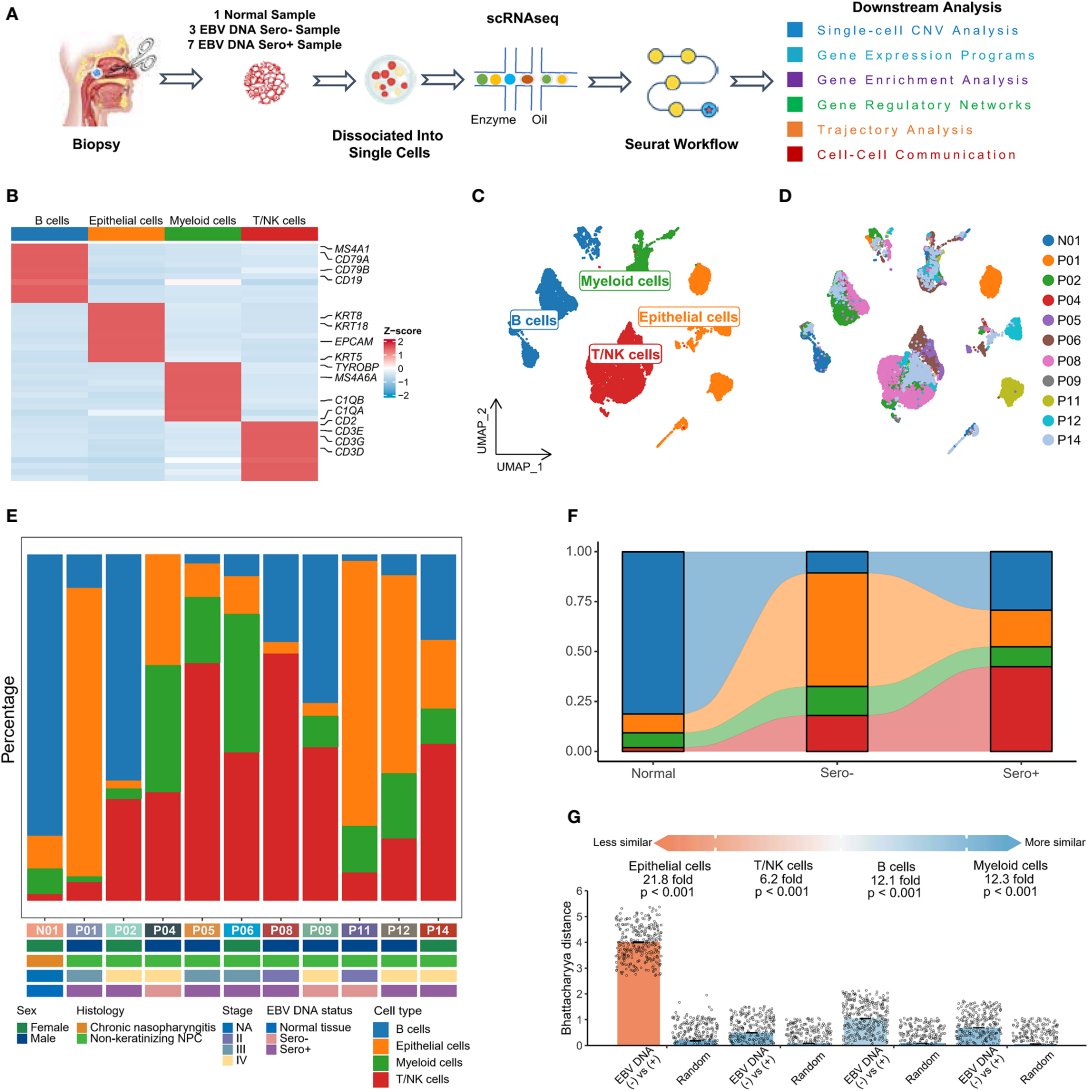
The landscape profiling of single cells in EBV DNA Sero- and Sero+ NPCs. **(A)** Schematic representation of the overall study design. **(B)** Classical cell markers were used to annotate major cell types as represented in the heatmap plot. **(C)** UMAP plot of single cells was classified into four major cell types. **(D)** UMAP plot of the above single cells was colored according to sample origin. **(E)** The fraction of each major cell type across 11 samples, along with clinical characteristics of each sample as indicated. **(F)** The fraction of each major cell type according to their origins from normal tissue, EBV DNA Sero- or Sero+ samples. **(G)** Quantification of differences in transcriptional profiles between cell types in EBV DNA Sero- and Sero+ samples using the Bhattacharyya distance. Each dot is a sub-sampling of 300 cells in principal component analysis space for EBV DNA Sero- and Sero+ samples or a random sampling of 300 cells independent of sample type. The height of the bar represents the mean of the sub-samples. P values are calculated with 100 replicates using a Wilcoxon rank-sum test comparing the EBV DNA Sero- *vs*. Sero+ cell types against the random selection for each cell type.

We obtained single-cell transcriptome sequencing data from ten NPC samples and one non-tumor nasopharyngeal tissue for bioinformatics analysis (**Figure 1A**, **Table S1, S2**). The ten NPC samples comprised three EBV DNA Sero- and seven EBV DNA Sero+ NPCs. After data processing and quality control (**Figure S1**), a total of 28,423 single cells were subsequently analyzed. We conducted major cell-type annotation based on classical gene markers (**Figure 1B**). Four major cell types were identified: epithelial cells, T/NK cells, B cells, and myeloid cells (**Figures 1C, D**). The cell composition in each sample, along with patient characteristics, are provided in **Figure 1E**. The distribution of these cell types differed significantly between the EBV DNA Sero- and Sero+ NPC groups (**Figure 1F**). The proportions of epithelial cells and myeloid cells were higher in the EBV DNA Sero- group while those of T/NK cells and B cells were higher in the EBV DNA Sero+ group. We then evaluated differences in transcriptional profiles between major cell types in EBV DNA Sero- and Sero+ groups using the Bhattacharyya distance (**Figure 1G**). Epithelial cells had the most considerable differences. The mean fold change between groups and random samples varied from 21.8-fold (epithelial cells) to 6.2-fold (T/NK cells). The above data revealed significant shifts in cellular compositions and transcriptomics between EBV DNA Sero- and Sero+ NPCs, suggesting a distinct TME context may be linked to EBV DNA seropositivity.

### 3.2 Complex clonal evolution of EBV-associated NPC malignant cells

**Figure 2:**
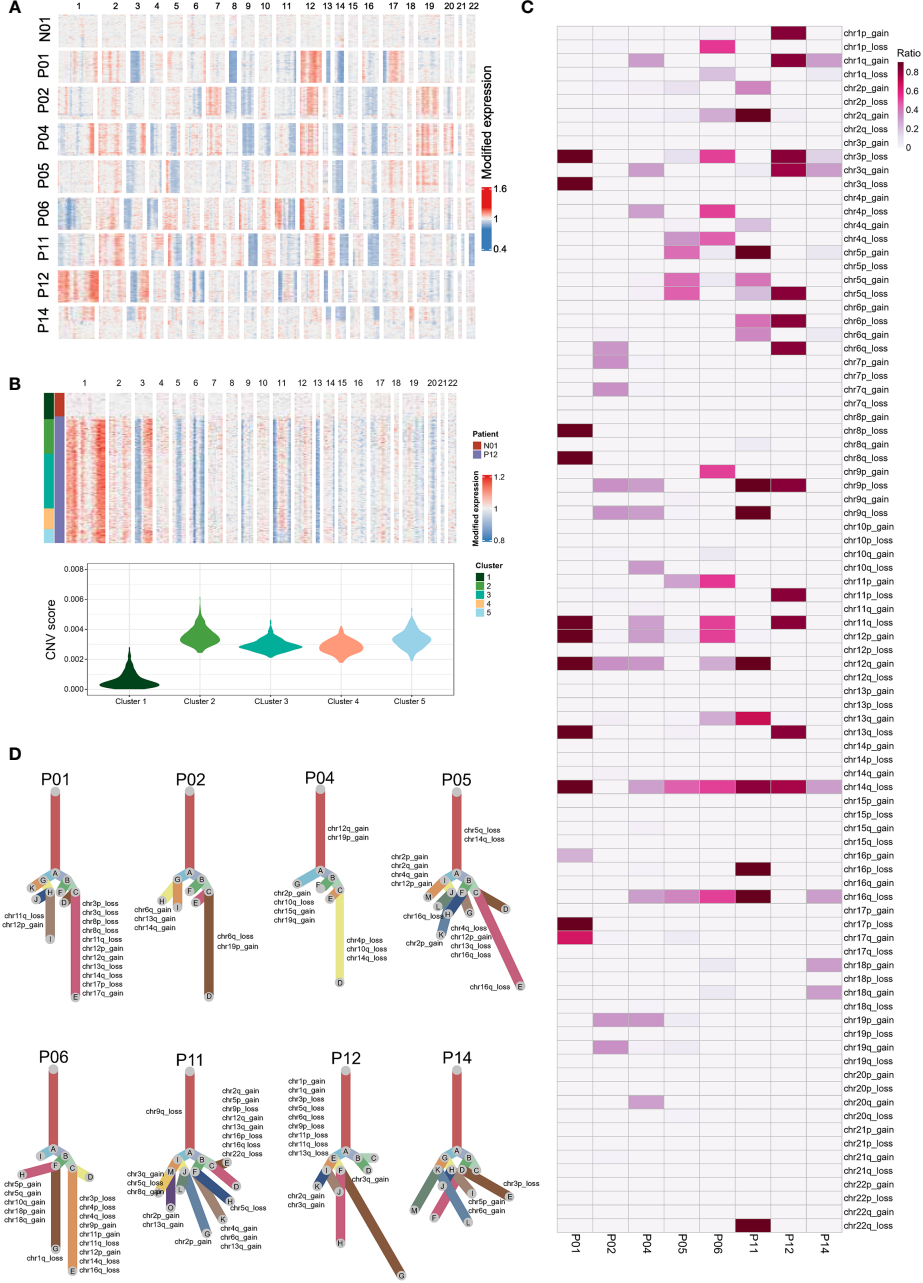
Single-cell copy-number variation (CNV) and clonality analysis. **(A)** Heatmap showed the large-scale CNVs of epithelial cells from one non-tumor tissue and eight NPC tumors. CNVs were inferred based on scRNA-seq data. **(B)** K-means clustering analysis was performed on epithelial cells derived from tumor samples (e.g., P12) and the non-tumor sample according to CNV scores (top panel). Violin plot showed the CNV scores for each cluster derived from K-means clustering (bottom panel). Epithelial cells of the tumor samples in the cluster (cluster 1) that predominantly contained cells from the normal sample were regarded as non-malignant cells. In contrast, the cells with high CNV scores in other clusters were identified as malignant cells. **(C)** The summary CNV profiles of malignant epithelial cells from each of the eight patients. CNVs were categorized by the chromosome arm and labeled as gain or loss in single cells. Color in the heatmap represented the ratio of the corresponding CNV events in the single cells from each individual sample. **(D)** Evolutionary trees of the single cells from each of the eight tumor samples. The tree branches are delineated according to the percentage of cells in the subclone containing the corresponding CNVs. The typical CNVs in each sample were labeled in the clonality tree. These trees were further modified manually using Adobe Illustrator for better layout following the developer’s suggestion.

We next analyzed eight NPC samples that each sample contained at least 50 epithelial cells from the individual sample, including two EBV DNA Sero- and six EBV DNA Sero+ samples. We used large-scale CNVs to distinguish the malignant cells from the non-malignant cells and probe the clonal structure of each tumor (**Figures 2A, B**). We identified 5,726 malignant cells from eight NPC samples for further analyses, The inferred CNV profiles indicated that deletions in chromosomes 3p, 9p, 11q, 14q, and 16q and amplifications in chromosomes 12q were frequent in NPC (**Figure 2C**), which were consistent with previously reported whole-exome sequencing results (31, 32). The clonality analysis revealed the complexity of typical and atypical CNV events in NPC (**Figure 2D**). Typical CNV events dominate the chromosomal landscape. Interestingly, we also observed multiple subclonal typical and atypical CNV events in some samples. This result indicated that canonical CNV events do not constantly occur in a single CNV event but can be generated in an evolving genome. Several previous studies have reported that tumors can evolve with the development of unknown atypical CNV subclones, which may contribute to tumor progression (24, 33).

### 3.3 Heterogeneity and differentiation trajectory of malignant cells from EBV DNA Sero- and Sero+ NPCs

**Figure 3:**
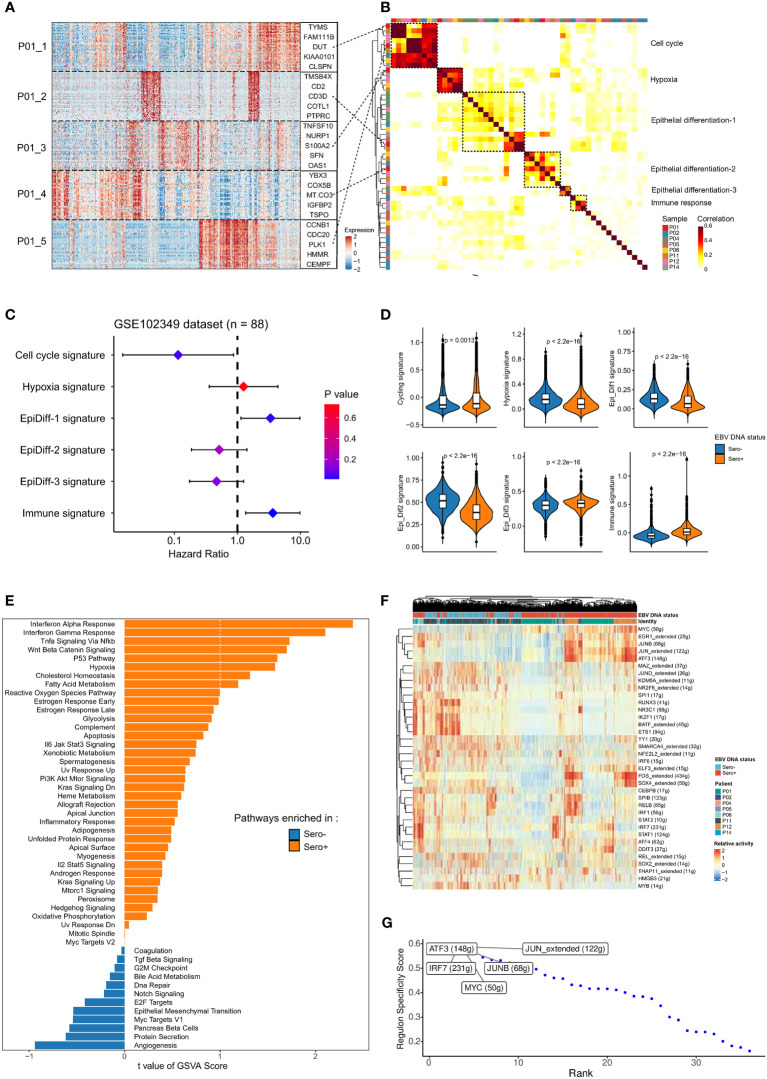
Malignant cells profiles between EBV DNA Sero- and Sero+ NPCs. **(A)** Heatmap shows the six gene expression programs decoded from a representative tumor (P01) using non-negative matrix factorization algorithm, along with the top 5 genes marked. **(B)** Heatmap depicts the pairwise correlations of 50 intra-tumor gene expression programs derived from 8 tumors. The correlation clustering identified six co-expression modules across the tumors. **(C)** Associations of the six co-expression modules and progression-free survival were evaluated in an NPC cohort (GSE102349, n = 88) and summarized in the forest plot. **(D)** Comparisons in gene expression for the six co-expression modules according to the EBV DNA status. P values were calculated using the Wilcoxon rank-sum test. **(E)** GSVA analysis reveals enriched hallmark pathways in EBV DNA Sero- *vs*. Sero+ malignant cells. **(F)** Heatmap shows the activity of transcription factors (TFs) in malignant cells derived from EBV DNA Sero- *vs*. Sero+ samples. **(G)** EBV DNA Sero+ sample-specific TFs were ranked by the Regulon Specificity Score. The top 5 activated TFs were indicated.

We next investigated the intertumor and intratumor heterogeneity of malignant NPC cells. We first used cNMF to identify a total of 50 gene expression programs that were preferentially co-expressed by subpopulations of malignant cells within each tumor across all samples (**Figure 3A**). Correlation clustering combined with GSEA was used to characterize these meta-programs into underlying co-expressed gene modules. We identified six co-expression modules (**Figure 3B**). We then analyzed the relationship between gene signatures of the modules and survival outcomes in bulk RNA-seq data of an NPC cohort (GSE102349, n = 88). Cell cycle signature, Epi-Diff 1 signature, and immune-related signature were significantly associated with progression-free survival (**Figure 3C**). The co-expressed gene modules were differentially expressed in EBV DNA Sero- and Sero+ NPC cells (**Figure 3D**). Specifically, NPC cells from EBV DNA Sero+ samples have higher cell cycle scores indicating higher proliferative capacity. Differential gene expression analysis showed that CDKN2A, ATF3, CXCL10, and interferon (IFN) stimulated genes (IFI6, IFIT3, IFI44L, IFITM1, etc.) were significantly upregulated in EBV DNA Sero+ NPC cells (**Figure S2**), suggesting more active EBV viral activity. We further used GSVA to reveal cancer hallmarks pathways. Notably, the results exhibited more cancer hallmarks pathways enriched in EBV DNA Sero+ samples, including IFN α/γ response, hypoxia, p53, and TNF-α signaling *via* NFκ-b pathways (**Figure 3E**). Nevertheless, a set of transcription factors (TFs) was upregulated in EBV DNA Sero+ tumor cells, evidenced by SCENIC analysis (**Figure 3F**). Among them, the expression of genes regulated by ATF3, JUN, IRF7, MYC, and JUNB showed highest regulon activity (**Figure 3G**).

**Figure 4:**
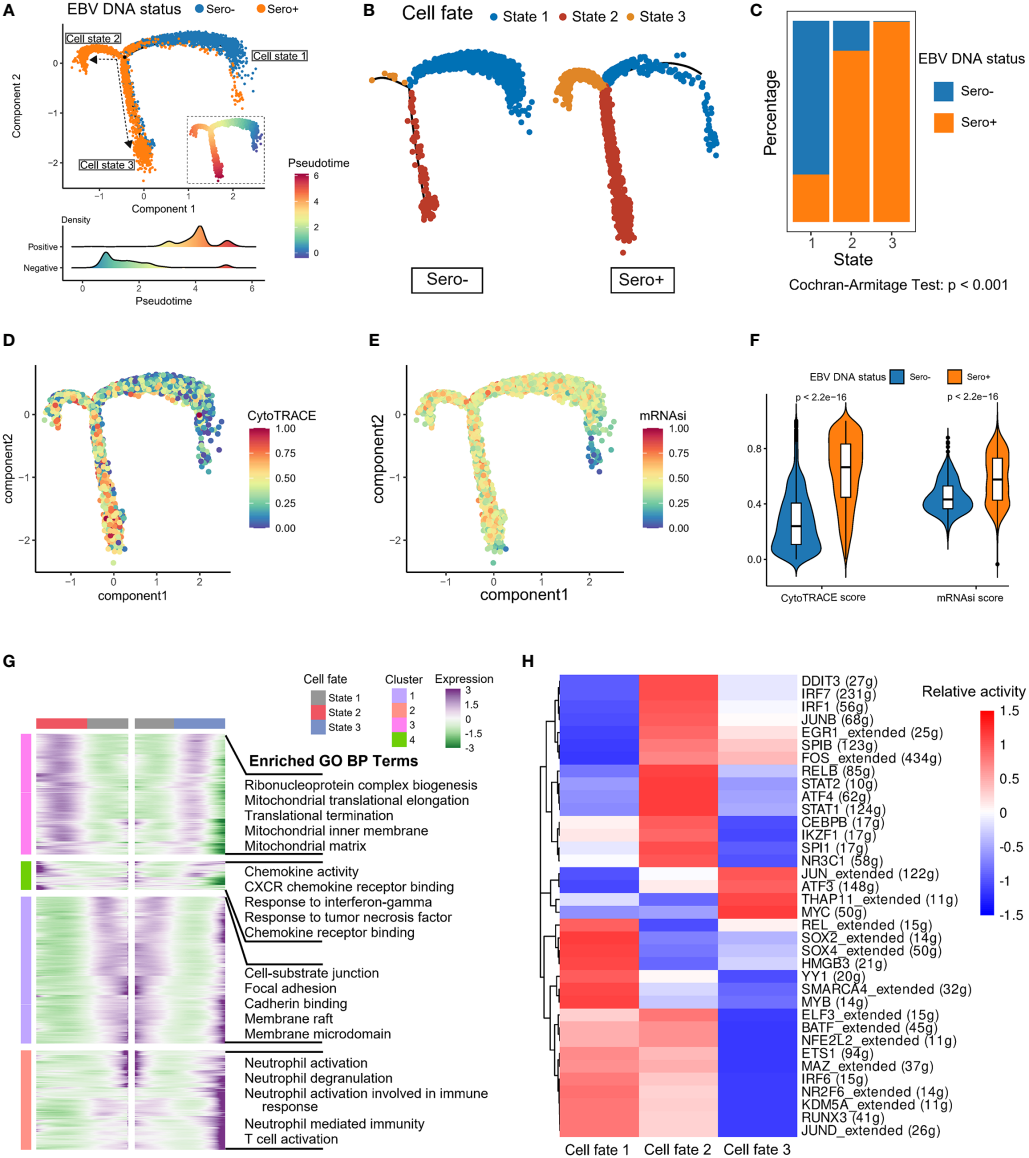
Development trajectory of malignant cells from EBV DNA Sero- and Sero+ NPCs. **(A)** Development trajectory of malignant cells colored by EBV DNA status (upper panel). Each cell was colored from blue to red and scaled by pseudotime scores (inlet panel). The cell density distribution of the pseudotime-ordered malignant cells from EBV DNA Sero- and Sero+ samples (lower panel). **(B)** Development trajectory of malignant cells derived from EBV DNA Sero- samples (left panel) and Sero+ samples (right panel). Cells were colored by cell states. **(C)** Bar graph shows the percentages of EBV DNA Sero- and Sero+ malignant cells across different cell states. P value was calculated by the Cochran-Armitage Test. **(D)** Development trajectory of malignant cells colored by the CytoTRACE scores. **(E)** Development trajectory of malignant cells colored by the mRNAsi scores. **(F)** Violin plot shows the expression levels of the CytoTRACE and mRNAsi scores between EBV DNA Sero- and Sero+ malignant cells. P values were calculated using the Wilcoxon rank-sum test. **(G)** Radiation heatmap shows the lineage-dependent gene expression patterns along the transformation of cell fate. **(H)** Heatmap showing the activated transcription factors in malignant cells across different cell states.

We next explored the dynamic transitions of NPC cells by inferring the developmental trajectories using Monocle2 (**Figure 4A**). The pseudotime analysis showed that EBV-associated NPC cells experienced three potential differentiation states. The distribution of cell states in tumor cells with different EBV DNA statuses varied significantly, that the EBV DNA Sero- tumor cells predominated at the beginning of the trajectory path (state 1), whereas most EBV DNA Sero+ tumor cells were at a terminal state (state 3) (**Figures 4B, C**). In addition, we used the CytoTRACE algorithm and OCLR algorithm to quantify the developmental potential and stemness index of tumor cells, respectively (**Figures 4D, E**). EBV DNA Sero+ NPC cells showed low-differentiation potential and higher stemness index than EBV DNA Sero- NPC cells (**Figure 4F**), which indicated the high aggressiveness of EBV DNA Sero+ NPC. Next, we visualized modules of genes that shared similar lineage-dependent expression patterns using the radiation heatmap (**Figure 4G**). The result indicated that cell states 2 and 3 had more such genes related to ribonucleoprotein complex, mitochondrial translation, and translational termination pathways. Besides, neutrophil and T cell-mediated immunity pathways were upregulated in the differentiation trajectory of cell state 3. Furthermore, the SCENIC analysis revealed that STAT1/2, ATF4, JUNB, IRF1/7, and other TFs related to cell signal transduction and IFN response pathways were highly activated in cell state 2, while cell state 3 had upregulated TFs of THAP11, MYC, ATF3, and JUN that related to cellular stemness, stress response, and cellular senescence pathways (**Figure 4H**).

The above data revealed significant alterations in transcriptomics and differentiation between EBV DNA Sero- and Sero+ NPC malignant cells, suggesting EBV seropositivity is strongly associated with NPC malignant cell development.

### 3.4 Transcriptional heterogeneity and dynamics in T cells are linked to EBV DNA seropositivity status

**Figure 5:**
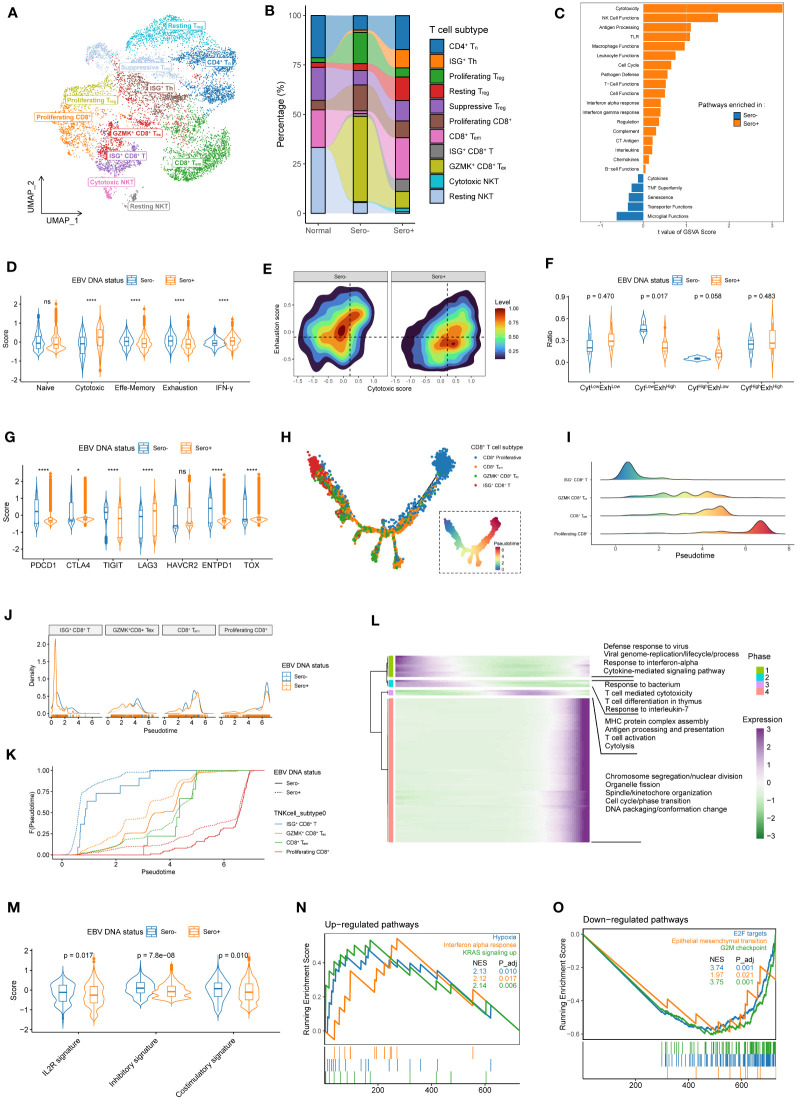
Transcriptional heterogeneity and dynamics in T cells are linked to EBV DNA status. **(A)** UMAP plot of T cells was grouped into 11 cell subtypes and indicated by color. **(B)** The proportion of 11 T cell subtypes in different sample origins. **(C)** GSVA analysis of enriched cancer immune pathways in CD8+ T cells derived from EBV DNA Sero- *vs*. Sero+ samples. **(D)** Violin plots shows the expression levels of the naïve, cytotoxic, effector memory, exhaustion, and IFN-γ signatures in EBV DNA Sero- and Sero+ CD8+ T cells. **(E)** Two-dimension density plot of the cytotoxicity and exhaustion states in EBV DNA Sero- and Sero+ CD8+ T cells. Cells are partitioned into four functional subtypes, including CytLowExhLow, CytLowExhHigh, CytHighExhLow, and CytHighExhHigh stats. **(F)** Violin plots shows the ratios of the four functional CD8+ T cell subtypes in EBV DNA Sero- and Sero+ CD8+ T cells. **(G)** Violin plot shows the expression levels of inhibitory markers in EBV DNA Sero- and Sero+ CD8+ T cells. **(H)** Development trajectory of CD8+ T cell subtypes. Each cell was colored from blue to red, scaled by pseudotime score (inlet panel). **(I)** The cell density distribution of the pseudotime-ordered CD8+ T cell subtypes. The area under the density curves was colored from blue to red and scaled by pseudotime scores. **(J)** The cell density distribution of the pseudotime-ordered CD8+ T cell subtypes in EBV DNA Sero- and Sero+ samples. **(K)** Cumulative distribution function shows the distribution of pseudotime-ordered CD8+ T cell subtypes in EBV DNA Sero- and Sero+ samples. A rightward shift of the curve indicates increased pseudotime scores. **(L)** The differentially expressed gene along the pseudotime were hierarchically clustered into four subclusters. The enriched GO terms in each cluster were indicated. **(M)** Violin plot shows the expression levels of IL2R, inhibitory, and costimulatory signatures in EBV DNA Sero- and Sero+ CD4+ T cells. **(N)** GSEA shows that the significantly up-regulated pathways in the EBV DNA Sero+ CD4+ T cells. **(O)** GSEA shows that the significantly down-regulated pathways in the EBV DNA Sero+ CD4+ T cells.

A total of 10,039 T/NK cells were analyzed and grouped into 16 clusters. Based on the DEGs (**Figure S3A**) and function-related markers (**Figure S3B**), the 16 clusters were classified into four CD8^+^ T cell subtypes (Cluster 12/13: proliferating T cells; Cluster 1/14: effector memory T cells [T_em_ cells]; Cluster 7/10: granzyme K-positive [GZMK^+^] exhausted T cells [T_ex_ cells]; Cluster 6: IFN-stimulated genes-positive [ISG^+^] T cells), five CD4^+^ T cell subtypes (Cluster 3/5: naive T cells [T_n_ cells]; Cluster 4: ISG^+^ T helper cells [T_h_ cells]; Cluster 9: proliferating regulatory T cells [T_reg_ cells]; Cluster 2: resting T_reg_ cells; Cluster 8/11: suppressive T_reg_ cells), and two natural-killer T (NKT) cell subtypes (Cluster 15: cytotoxic NKT cells; Cluster 16: resting NKT cells) (**Figure 5A**). The proportion of T cell subtypes differed among patients (**Figure S3C**) and patients with different EBV seropositivity status (**Figure 5B**).

The overall pathway analysis in CD8^+^ T cells with GSVA revealed that pathways of cytotoxicity, NK cell functions, antigen processing, and Toll-like receptors were upregulated in EBV DNA Sero+ CD8^+^ T cells, which is consistent with activated immune defendant machinery to fight against EBV activation (**Figure 5C**). Remarkably, cytotoxic and IFN-γ signatures were elevated in EBV DNA Sero+ samples, while naïve, effector memory, and exhaustion signatures were elevated in EBV DNA Sero- samples (**Figure 5D**). According to the cytotoxic and exhausted signatures (**Figure 5E**), CD8^+^ T cells were further grouped into four functional subtypes (Cyt^Low^Exh^Low^, Cyt^Low^Exh^High^, Cyt^High^Exh^Low^, and Cyt^High^Exh^High^). EBV DNA Sero+ group had more Cyt^Low^Exh^High^ CD8^+^ T cells and fewer Cyt^High^Exh^Low^ CD8^+^ T cells (**Figure 5F**), suggesting immunosuppressive TME associated with EBV DNA seropositivity status. In addition, certain classical inhibitory markers, such as CTLA4, LAG3, and HAVCR2 were upregulated EBV DNA Sero- CD8^+^ T cells, whereas other inhibitory markers, such as PDCD1, TIGHT, ENTPD1, and TOX were upregulated in EBV DNA Sero- CD8^+^ T cells (**Figure 5G**). These indicated different immunoinhibitory mechanisms employed by malignant cells depending on EBV DNA seropositivity status and may be helpful to guide the future development of immunotherapy strategy based on EBV seropositivity status.

In comparing cell composition, ISG^+^ CD8^+^ T and CD8^+^ T_em_ levels were elevated in EBV DNA Sero+ samples, whereas GZMK^+^ CD8^+^ T_ex_ and proliferating CD8^+^ T levels were elevated in EBV DNA Sero- samples (**Figure S3D**). We further explored the dynamic differentiation lineages and cell transitions of these CD8^+^ T cell subtypes by inferring the pseudotime trajectories (**Figure 5H**). This analysis showed a developmental trajectory that mainly began with the ISG^+^ CD8^+^ T cells, bifurcated into either the CD8^+^ T_em_ cells or the GZMK^+^ CD8^+^ T_ex_, and ended with proliferating CD8^+^ T cells (**Figure 5I**). CD8^+^ T cell subtypes showed relatively similar distribution patterns along with the pseudotime in samples with different EBV DNA statuses, except that ISG^+^ CD8^+^ T cells exhibited a peaked distribution at the very beginning (**Figure 5J**). Overall, EBV DNA Sero+ CD8^+^ T cells presented a lower pseudotime score compared with EBV DNA Sero- CD8^+^ T cells (**Figure 5K**). These results indicated that the cytotoxic T-lymphocyte response may be triggered early in EBV DNA Sero+ NPC. We next examined the transcriptional changes related to the differentiation process and found that the CD8^+^ T cell differentiation lineages could be classified into four major phases with distinct enriched GO pathways (**Figure 5L**).

We next examined the cell composition and transcriptome heterogeneity of CD4^+^ T cells driven by EBV DNA status. Overall, the EBV DNA Sero+ group had a higher proportion of T_reg_ cells (53.3 *vs*. 35.0%) than the EBV DNA Sero- samples. The EBV DNA Sero+ group had more ISG^+^ Th cells (17.3% *vs*. 3.4%) and resting T_reg_ cells (22.4% *vs*. 9.8%) but fewer proliferating T_reg_ cells (8.7% *vs*. 45.1%) than that in EBV DNA Sero- group. A recent report suggested that ISG^+^ Th cells might represent a fraction of T_reg_ cells that responded to type I IFNs in TME during activation (34). The total IL2R, inhibitory, and costimulatory signatures were all elevated in the EBV DNA Sero- CD4^+^ T cells (**Figure 5M**). The result suggested that T_reg_ cells were more activated in EBV DNA Sero- NPC and had a more immunosuppressive context than the EBV DNA Sero+ NPC. GSEA showed that the hypoxia, IFN-α response, and KRAS signaling were significantly upregulated in the EBV DNA Sero+ samples (**Figure 5N**), whereas E2F targets, epithelial-mesenchymal transition, and G2M checkpoint were upregulated in the EBV DNA Sero- samples (**Figure 5O**).

### 3.5 Diversity and transcriptional heterogeneity in B cells

**Figure 6:**
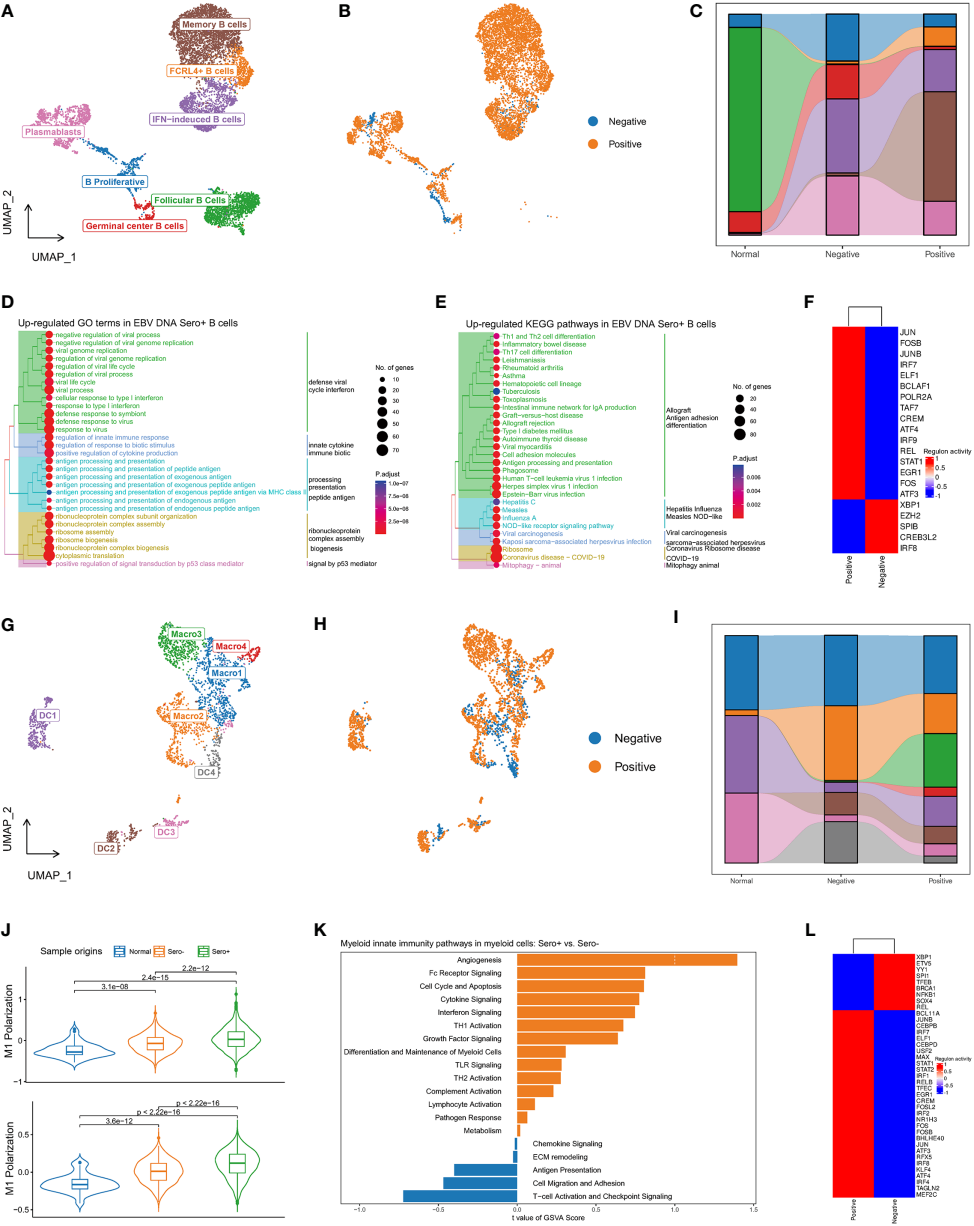
Diversity and transcriptional heterogeneity in B and Myeloid cells. **(A)** UMAP plot of B cells was grouped into 7 cell subtypes and indicated by color. **(B)** UMAP plot of B cells colored by EBV DNA status. **(C)** The proportion of 7 B cell subtypes in different sample origins. **(D)** GSEA analysis reveals up-regulated GO terms in EBV DNA Sero+ B cells. **(E)** GSEA analysis reveals up-regulated KEGG pathways in EBV DNA Sero+ B cells. **(F)** Heatmap shows the activated transcription factors in EBV DNA Sero- and Sero+ B cells. **(G)** UMAP plot of myeloid cells was grouped into eight cell subtypes and indicated by color. **(H)** UMAP plot of myeloid cells colored by EBV DNA status. **(I)** The proportion of eight myeloid cell subtypes in different sample origins. **(J)** Violin plot shows the expression levels of M1 and M2 polarization scores in EBV DNA Sero+ macrophages. **(K)** GSVA analysis reveals enriched myeloid innate immunity pathways in EBV DNA Sero- *vs*. Sero+ myeloid cells. **(L)** Heatmap shows the activated transcription factors in EBV DNA Sero- and Sero+ myeloid cells.

In total, 8592 B cells were identified and grouped into seven subsets according to the DEGs and classic markers (**Figures S4A, B**, **Figure 6A**), including memory B cells (cluster 1), follicular B cells (cluster 2), IFN-induced B Cells (cluster 3), plasmablasts (cluster 4/7), FCRL4+ B cells (cluster 5), proliferating B cells (cluster 6), and germinal center B cells (cluster 8). The proportion of B cell subtypes showed variation across patients (**Figure S4C**) and between groups with different EBV DNA statuses (**Figures 6B, C**). The proportions of FCRL4+ B cells and memory B cells were higher in the EBV DNA Sero+ samples, whereas that of the proliferating B cells and germinal center B cells were higher in the EBV DNA Sero- samples, indicating a strong immunoregulatory potential in EBV DNA Sero+ NPC. GO enrichment analysis showed that the antiviral response, IFN response, antigen processing and presentation, and ribosome assembly were activated in the EBV DNA Sero+ group (**Figure 6D**). KEGG pathway enrichment analysis revealed strong activation of T-cell differentiation, antigen processing and presentation, and EBV infection among EBV DNA Sero+ samples (**Figure 6E**). SCENIC analysis revealed that TFs of JUN/JUNB, FOS/FOSB, IRF7/9, ELF1, STAT1 were upregulated in EBV DNA Sero+ samples (**Figure 6F**). These observations indicate that B cells in EBV DNA Sero+ NPC exhibit an inflammation-dominant antiviral immune response.

### 3.6 Diversity and transcriptional heterogeneity in myeloid cells

The re-clustering of 2,902 myeloid cells revealed eight populations (**Figure 6G**), including four subsets of macrophages and four subsets of dendritic cells (DCs) (**Figures S5A, B**). We could not distinguish M1 and M2 macrophages based on curated marker genes, however, we examined the expression of M1 and M2 polarization signatures of macrophages subsets (**Figure S5C**). Macro1 was a classic activated M1 macrophage with high activation of IL1B, IL8, LYZ, and IFI30 genes, characterized by intermediate M1 polarization score and low M2 polarization score. Macro2 was a proliferating macrophage marked with high expression of cell cycle-related genes and low M1 and M2 polarization scores. Macro3 showed high M1 and M2 polarization signals, consistent with previous reports on M1/M2 couple-activated macrophages in NPC (16, 35). Macro4 showed a high M2 polarization signal, which may be a selectively activated M2 macrophage. DC1 was plasmacytoid DCs (pDCs) characterized by increased expression of CLEC4 and IGJ genes. DC2 was a mature DC that showed high expression levels of LAMP3 and CCR7, yielding a high potential for migration, activation, and maturation (**Figure S5D**). DC3 represented the classical DCs that were characterized by overexpression of CLEC9A. DC4 showed increased expression of CD1A, CD207, and ID2, suggesting that these cells were Langerhans cells (LCs). The myeloid cell compositions differed among patients (**Figure S5E**) and between EBV DNA Sero- and EBV DNA Sero+ samples (**Figures 6H, I**). Notably, Macro3 (99.4%) and Macro4 (98.9%) were almost all from EBV DNA Sero+ samples, suggesting predominant macrophage activation and M2 polarization. In the meanwhile, the M1 and M2 polarization scores were significantly upgraded in EBV DNA Sero+ NPC (**Figure 6J**). GSVA suggested different patterns of enriched pathways in the myeloid compartment between different EBV DNA statuses. Several pathways involved in myeloid innate immunity were upregulated in EBV DNA Sero+ myeloid cells, including angiogenesis, Fc receptor signaling, cell cycle and apoptosis, cytokine signaling, and IFN signaling pathway (**Figure 6K**). In contrast, T cell activation and immune checkpoint signaling, cell migration adhesion, and antigen presentation signaling pathways were activated in EBV DNA Sero- myeloid cells. SCENIC analysis revealed that TFs of JUN/JUNB, FOS/FOSB/FOSL2, IRF1/2/4/7/8, and STAT1/2 were upregulated in EBV DNA Sero+ samples (**Figure 6L**). These observations indicated that the ability to shape the immune environment was enhanced in EBV DNA Sero+ NPC.

### 3.7 Global up-regulation of IFN responses and enhanced cell communication in EBV DNA Sero+ NPC

**Figure 7:**
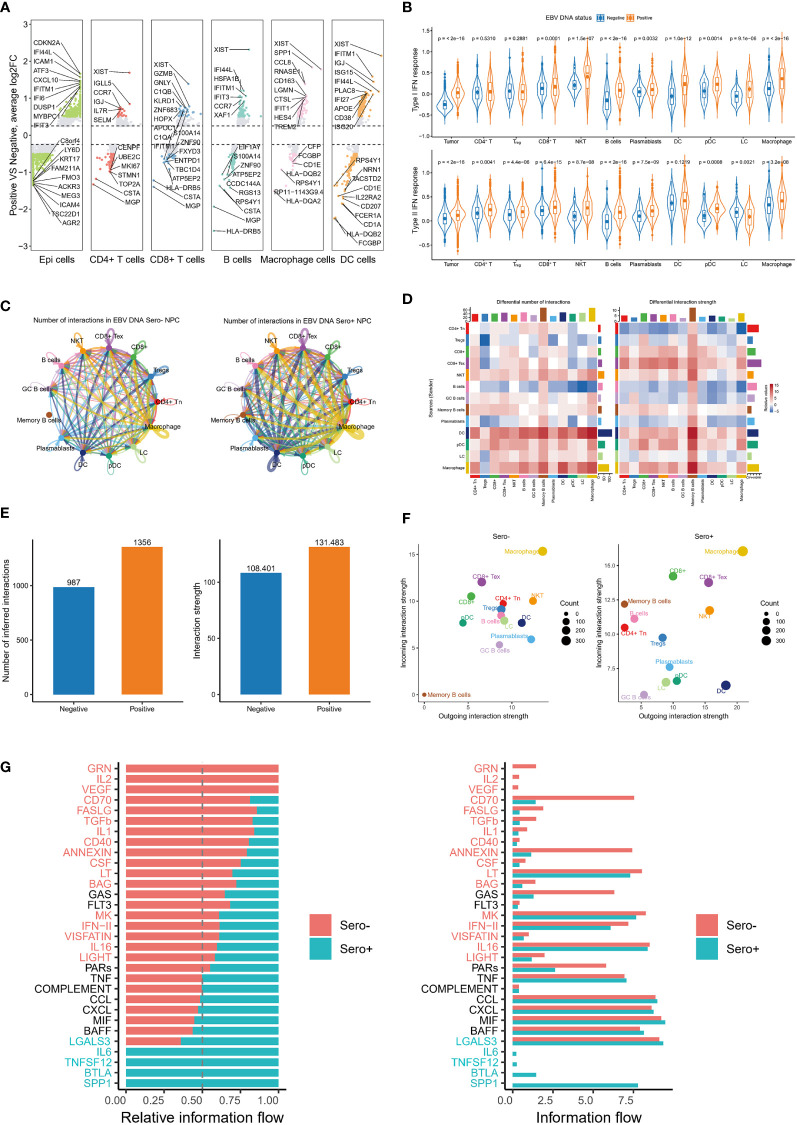
Global up-regulation of IFN responses and enhanced cell communication in EBV DNA Sero+ NPC. **(A)** Differentially expressed gene analysis reveals significantly up-regulated and down-regulated genes in six major cell types in EBV DNA Sero- and Sero+ samples. **(B)** Violin plot shows the type I IFN response and type II IFN response scores across cell subtypes in in EBV DNA Sero- and Sero+ samples. **(C)** Circle plot shows the differential number of interactions or interaction strength in the cell-cell communication network between EBV DNA Sero- and Sero+ samples. Red (or blue) colored edges represent increased (or decreased) signaling in the EBV DNA Sero+ samples compared to EBV DNA Sero- samples. **(D)** Heatmap shows differential number of interactions or interaction strength. The colored bar plot at top represents the total incoming signaling across different cell types. The right colored bar plot represents the total incoming signaling across different cell types. In the color bar, red (or blue) represents increased (or decreased) signaling in EBV DNA Sero+ samples compared to EBV DNA Sero- samples. **(E)** The bar plot shows the total number of interactions and interaction strength in EBV DNA Sero- and Sero+ samples. **(F)** Scatter plot shows the outgoing and incoming interaction strength across different cell types in 2D space between EBV DNA Sero- and Sero+ samples. **(G)** Bar graph shows significant signaling pathways ranked based on differences in the overall information flow within the inferred networks between EBV DNA Sero- and Sero+ samples. The left bar graph was plotted in a stacked mode while the right one was not. The top signaling pathways colored red were enriched in EBV DNA Sero- samples, and these colored greens were enriched in the EBV DNA Sero+ samples.

Previous studies have reported that IFN responses were significantly upregulated in the TME of NPC, especially in recurrent NPC (16, 35). Our DEG analysis suggested that genes related to IFN responses were upregulated in most cell types of EBV seropositive NPC (**Figure 7A**). We further calculated type I IFN response and type II IFN response scores for each major cell type using well-defined gene markers. The results showed that type I IFN response and type II IFN response scores were significantly upgraded in almost all cell types from EBV DNA Sero+ samples (**Figure 7B**). These observations suggest that the IFN responses are globally upregulated in the TME of EBV DNA Sero+ NPC, exhibiting an antiviral immune response-dominated context.

We next used the CellChat package (version 1.1.3) to explore the cellular communication network in NPC immune microenvironment. We detected complex communications between immune cells (**Figure 7C**). Memory B cells, macrophages, and DCs showed the highest intercellular interactions. Overall, the number and intensity of cell-cell interactions in EBV DNA Sero+ NPC were higher than those in EBV DNA Sero- NPC (**Figures 7D, E**). Macrophages represented the cell type with the most incoming and outgoing signalings, regardless of EBV DNA status (**Figure 7F**). Surprisingly, we did not observe incoming and outgoing signalings in memory B cells from EBV DNA Sero- samples, while memory B cells from EBV DNA Sero+ samples showed strong incoming signalings, indicating a stronger immunoregulatory potential. We further identified and ranked the essential signaling pathways (**Figures 7G, H**). Several signaling pathways that induce inflammatory responses and are involved in immune regulation were significantly upregulated in EBV DNA Sero+ NPC, such as SPP1, BTLA, TNFSF12, and IL6. SPP1, as a cytokine, plays a vital role in the type-I immune response by enhancing the production of IFN-γ and IL12 and reducing the production of IL10.

## 4 Discussion

Our previous work has suggested that EBV DNA Sero- and Sero+ NPCs are distinctly different disease subtypes (8). Here, we move forward to compare the tumor ecosystems of EBV DNA Sero- and Sero+ NPCs at single-cell resolution. Our data revealed a complex multicellular ecosystem in EBV-associated NPC, including intratumoral and intertumor heterogeneity, diversity and dynamics of immune lineages, and potential cellular interaction network. Tumor cells from EBV DNA Sero+ NPC exhibited low-differentiation potential, stronger stemness, and upregulated signaling pathways associated with cancer hallmarks than that of EBV DNA Sero- NPC. The low expression of classical immune checkpoint, early-triggered cytotoxic T-lymphocyte response, global activation of IFN-mediated signatures, and enhanced cell-cell interplays cooperatively tend to form a specific immune context in EBV DNA Sero+ NPC.

EBV infection is closely related to malignant transformation and tumorigenesis of EBV-associated NPC (36). B cells and epithelial cells are generally considered to be the primary host of EBV (37). In this study, we found that pathways of EBV infection, antigen presentation, and antiviral response were significantly enriched in B cells from EBV DNA Sero+ samples, suggesting an active antiviral immune response. Previous studies have reported that EBV infection status can affect the transcriptions in NPC tumor cells. Pathways associated with EBV infection are activated explicitly in EBV-related NPC cells, such as NFκ-B and Notch pathways (38). Our data revealed that tumor cells with different EBV DNA seropositivity statuses had distinct transcriptional profiles and trajectories. Most cancer hallmark signaling pathways were significantly activated in tumor cells from EBV DNA Sero+ samples, such as IFN-mediated immune responses, P53 pathway, hypoxia, and inflammation-related pathways. Tumor cells derived from EBV DNA Sero+ samples also had low-differentiation potential and higher tumor stemness features, which have been proven to be poor prognostic features in various tumors (29, 30). Our trajectory analysis revealed signaling pathways activated differentially along with pseudotime, suggesting that the antiviral immune response may be gradually activated during the transdifferentiation of tumor cells.

In chronic infection and tumor microenvironment, CD8^+^ T cells gradually become exhausted due to long-term antigenic exposure and inflammatory stimulation (39). CD8+ T cell exhaustion is a gradual process characterized by decreased cytotoxicity and proliferation and increased expression of inhibitory markers (40). Preventing or reversing CD8^+^ T cell exhaustion and restoring its cytotoxicity are the key to improving the efficacy of immunotherapy in clinical practice (41). In this study, we observed a significant difference in the transcriptions and dynamics of T cells between EBV DNA Sero- and Sero+ NPCs. CD8^+^ T cells from EBV DNA Sero+ samples had higher cytotoxic activity and lower exhaustion levels, indicating a more robust immune response. We identified a CD8^+^ T subpopulation (GZMK^+^ CD8^+^ T_ex_) characterized by fair cytotoxicity and certain level of exhaustion, which seemed to be precursors of exhausted T cells (40). Previous studies have reported that elevated expression of cytotoxicity markers and inhibitory receptors in CD8^+^ T cells is positively associated with potential clinical benefits of anti-PD-1 therapy (42). These observations suggested that CD8^+^ T cells from EBV DNA Sero- samples, with certain exhaustion signatures while maintaining fair cytotoxicity, may respond better to anti-PD-1 treatment. Reversing the exhaustion state of CD8^+^ T cells and restoring their killing effect will be the key to improving the efficacy of immunotherapy further. Our data also showed that CTLA4, LAG3, and HAVCR2 were upregulated EBV DNA Sero- CD8^+^ T cells, while PDCD1, TIGHT, and ENTPD1 were upregulated in EBV DNA Sero- CD8^+^ T cells, which further supports the rationale of individualized immunotherapy targeting on NPC with different EBV DNA seropositivity status. Notably, combined immunotherapy targeting multiple inhibitory receptors has been investigated to enhance the efficacy, such as anti-PD-1 combined with anti-CTLA4 and anti-TIM3 combined with anti-LAG3 (40). Therefore, anti-PD-1 therapy combined with anti-CTLA4 or anti-LAG3 treatment may be a potential immunotherapy strategy for EBV DNA Sero+ NPC, which is expected to further improve the response rate in this high-risk population.

In the current study, we observed that IFN responses were upregulated in almost all cell types from EBV DNA Sero+ NPC. In addition to antiviral immune responses, IFN responses also play an essential role in adjusting cancer-related immune function. However, the IFN response is a double-edged sword in terms of the antitumor immune response (43–45). On the one hand, IFN response can drive immune activation, including inducing direct cell killing, stimulating antigen-presenting cells to improve tumor immunogenicity, and enhancing the cytotoxicity of CTL. On the other hand, IFN response promotes immunosuppressive effects, including mediating the inactivation of B cells to block antibody protective mechanisms (46, 47), inducing the expression of inhibitors and co-simulators to mediate tumor-promoting effects (45, 48, 49), and downregulating the expression of MHC class I molecules to evade immune surveillance (50). Whether IFN-induced signaling produces antitumor or pro-tumor effects depends mainly on the duration and intensity of the IFN response (43). However, the duration and intensity of IFN signaling is often controlled by tumor burden and immune cell infiltration status in TME. Therefore, we hypothesized that the sustained IFN responses in the TME of EBV DNA Sero+ NPC render tumor-driven adaptive immunity resistant, enhance the immunosuppressive capacity of tumor cells, and ultimately lead to malignant transformation and therapeutic resistance. Although IFN responses are the original mechanism to fight against EBV infection, consistent IFN responses after the malignant transformation of epithelial cells can result in shaping TME into a more tolerant state, promoting tumoral signal, facilitating tumor cell survival, and leading to adverse outcomes (51). Therefore, modulating IFN response in combination with ICIs may be a promising treatment strategy for EBV DNA Sero+ NPC. To date, several studies have reported preliminary results of improving the efficacy of tumor immunotherapy by modulating the IFN response (43, 44, 52–56). However, we still need to address several key questions, including which IFN-producing cells are the most important in fighting tumors, which IFN-producing cells mediate adaptive immune resistance, and how to fine tune the IFN response to enhance the efficacy of immunotherapy. Future studies must explore additional pathways to modulate IFN-mediated proinflammatory and anti-inflammatory effects to develop better therapeutic strategies to promote its antitumor capabilities and prevent immune escape (43).

We acknowledge that the current study has several limitations. First, regarding the limited sample size, a large patient cohort may help to validate our results further and reduce potential selection bias. Second, the only non-tumor nasopharyngeal tissue is not matched to these tumor samples but from a patient with chronic nasopharyngitis. A paired normal tissue can be the ideal control sample and minimizes the individual difference other than tumor itself. However, the paired non-tumor tissue is not easily accessible due to the primary treatment of NPC is radiotherapy but not surgery. There is still much uncertainty in obtaining the normal tissue during biopsy. Besides, in consideration of humanistic care, multi-site biopsy is not routine performed unless upon the clinical requirements. Third, although we have identified complex molecular compositions and close cellular interactions in TME through bioinformatic analysis, further cell function experiments and animal experiments will help elucidate the underlying biological mechanisms and draw more robust conclusions. Lastly, further integrated analyses of single-cell multi-omics, such as TCR/BCR analysis, single-cell DNA sequencing, single-cell proteomics, and spatial transcriptomics will help deepen our understanding of TME heterogeneity between NPC with different EBV DNA seropositivity status.

## 5 Conclusion

Taken together, the current scRNA-seq analysis deepened our understanding of TME heterogeneity in EBV-associated NPC and identified the potential cell lineages and interacting molecules that may contribute to immunosuppression and tumor progression. These findings provide important clues to elucidate the mechanisms of NPC tumorigenesis and to develop more effective immunotherapy strategies. Further experiments *in vitro* and *in vivo* are needed to explore the underlying biological mechanisms in the future.

## Data availability statement

Publicly available datasets were analyzed in this study. This data can be found here: The processed scRNA-seq dataset was deposited at the Gene expression omnibus (GEO) data repository under the accession code GSE150430. The bulk RNA-seq dataset of the NPC cohort could be downloaded from GEO under the accession numbers GSE102349. The remaining data are present in **Supplementary Materials**, or available from the authors upon reasonable request.

## Ethics statement

The studies involving human participants were reviewed and approved by the Institutional Review Board of Sun Yat-sen University Cancer Center. Written informed consent to participate in this study was provided by the participants’ legal guardian/next of kin.

## Author contributions

WL, YX, CX, and JH conceived and designed the study. WL analyzed the data and wrote the manuscript with inputs from all authors. All authors edited and revised the manuscript, provided comments, and coordinated the collaboration. WL, SL, GL, and NL contributed equally to this work and share first authorship. YX, CX, and JH contributed equally to this work and share last authorship. All authors contributed to the article and approved the submitted version.
