# Supplementary material for: Epstein-Barr virus DNA seropositivity links distinct tumoral heterogeneity and immune landscape in nasopharyngeal carcinoma
Source: Front Immunol. 2023 Feb 13;14:1124066. doi: 10.3389/fimmu.2023.1124066 (PMC9968721; doi:10.3389/fimmu.2023.1124066)
Supplement: Supplementary file 1 [file DataSheet_1.docx]

Supplementary Material

Epstein-Barr virus DNA seropositivity links distinct tumoral heterogeneity and immune landscape in nasopharyngeal carcinoma

Wangzhong Li ^1,2^^+^, Shuhui Lv^3+^, Guoying Liu^4+^, Nian Lu^2+^, Yaofei Jiang^2^, Hu Liang^2^, Weixiong Xia^2^, Yanqun Xiang^2*^, Changqing Xie^5*^, Jianxing He^1*^

*** Correspondence:**Jianxing He ([drjianxing.he@gmail.com](mailto:drjianxing.he@gmail.com)), Department of Thoracic Surgery and Oncology, The First Affiliated Hospital of Guangzhou Medical University, 151 Yanjiang Road, Guangzhou, 510120, China; Changqing Xie ([changqing.xie@nih.gov](mailto:changqing.xie@nih.gov)), Thoracic and Gastrointestinal Malignancies Branch, Center for Cancer Research, National Cancer Institute, National Institutes of Health, Bethesda, MD, United States; Yanqun Xiang ([xiangyq@sysucc.org.cn](mailto:xiangyq@sysucc.org.cn)), Department of Nasopharyngeal Carcinoma, Sun Yat-sen University Cancer Center, the State Key Laboratory of Oncology in South China, Collaborative Innovation Center for Cancer Medicine, Guangdong Key Laboratory of Nasopharyngeal Carcinoma Diagnosis and Therapy, 651 Dongfeng Road East, Guangzhou, China.

# Supplementary Figures and Tables

## Supplementary Figures


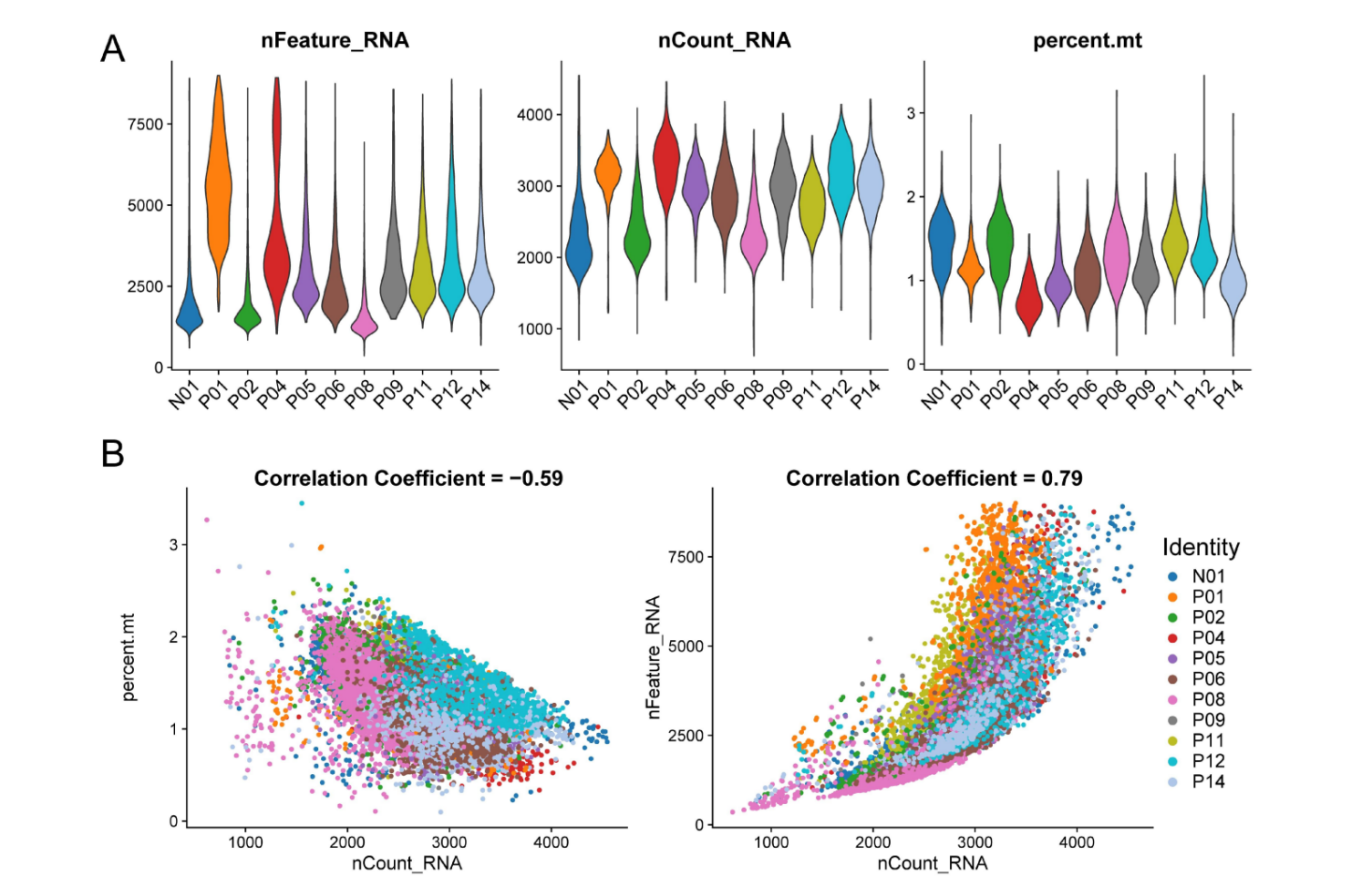


**Fig. S1** (A) Violin plot shows the levels of nFeature_RNA, nCount_RNA, and percent.mt across

11 samples after quality control. (B) Scatter plot shows the correlation between nFeature_RNA

and percent.mt (left panel) and the correlation between nCount_RNA and nFeature_RNA (right

panel). Each dot represents a single cell and colored by sample origin.


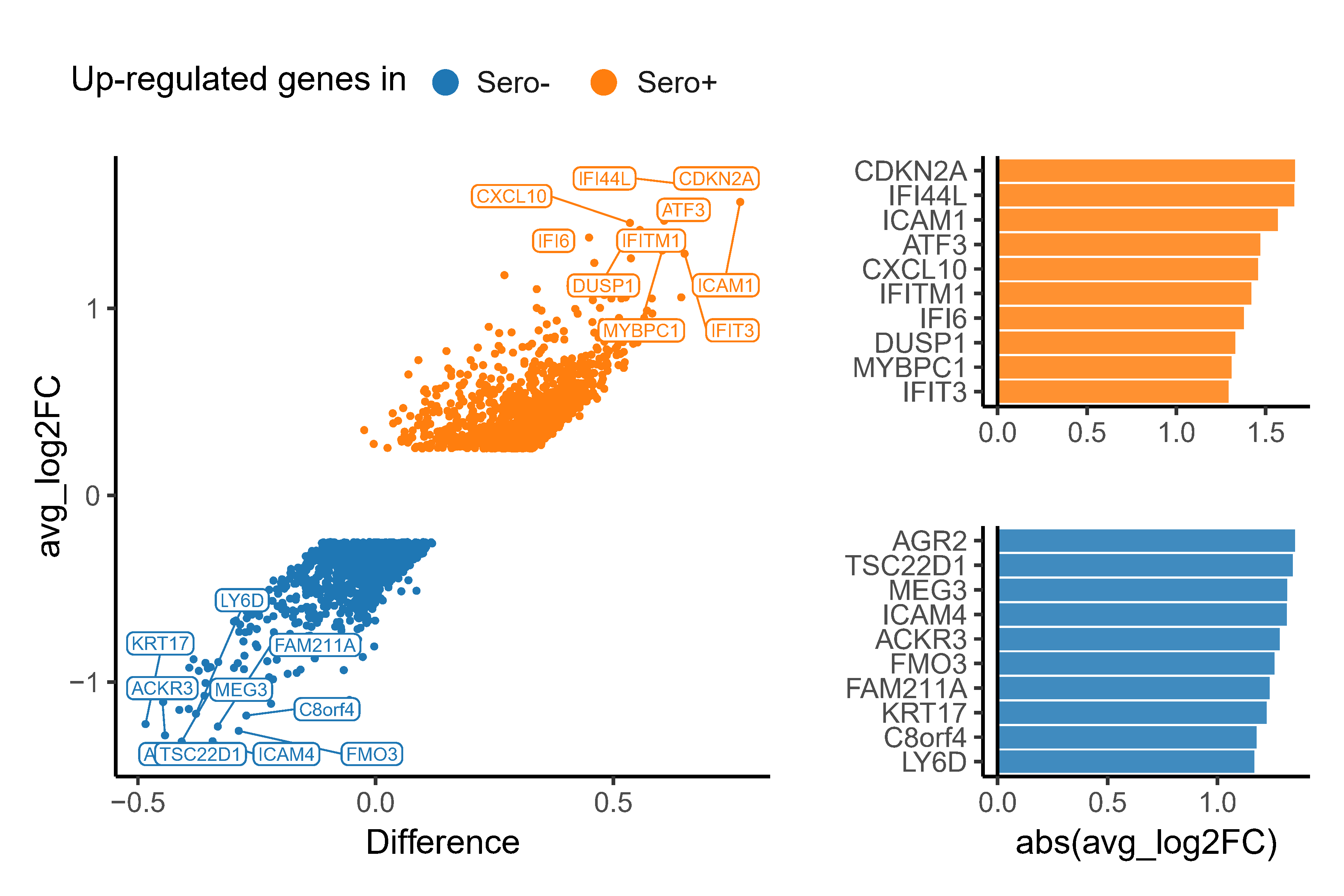


**Fig. S2** Differential gene expression analysis shows significantly up-regulated in EBV DNA

Sero- and Sero+ malignant NPC cells. The top 10 genes were provided along with absolute

average log-fold change values (right panel).


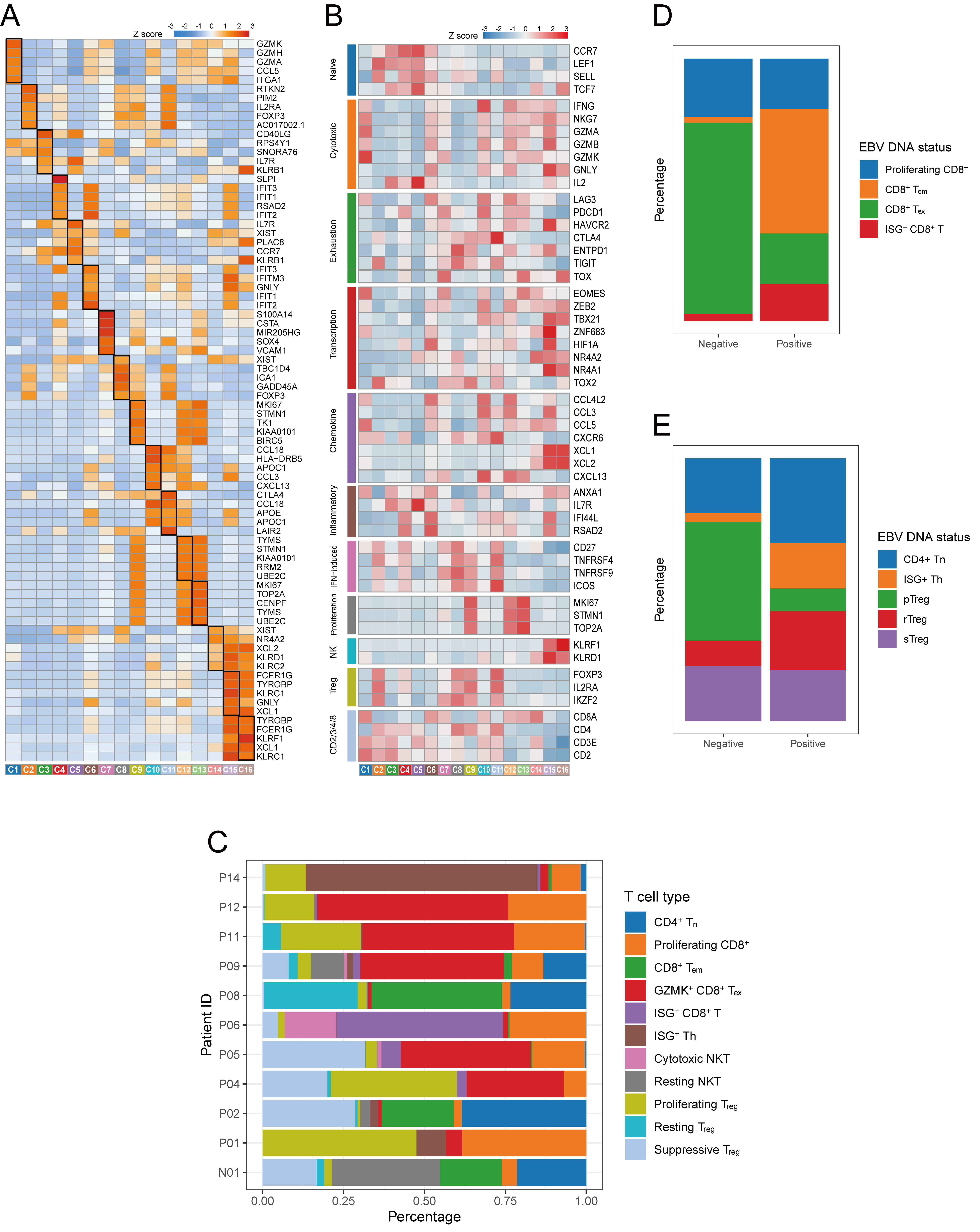


**Fig. S3** (A) Heatmap shows the top 5 differently expressed genes of each of 16 cluster of T/NK cells. (B) Heatmap shows the expression levels of conserved gene markers or functional gene markers in 16 T/NK cell subtypes. (C) The proportion of 11 T/NK cell subtypes in different sample origins. (D) The proportion of five CD4+ T cell subtypes in EBV DNA Sero- and Sero+ samples. (E) The proportion of five CD8+ T cell subtypes in EBV DNA Sero- and Sero+ samples.


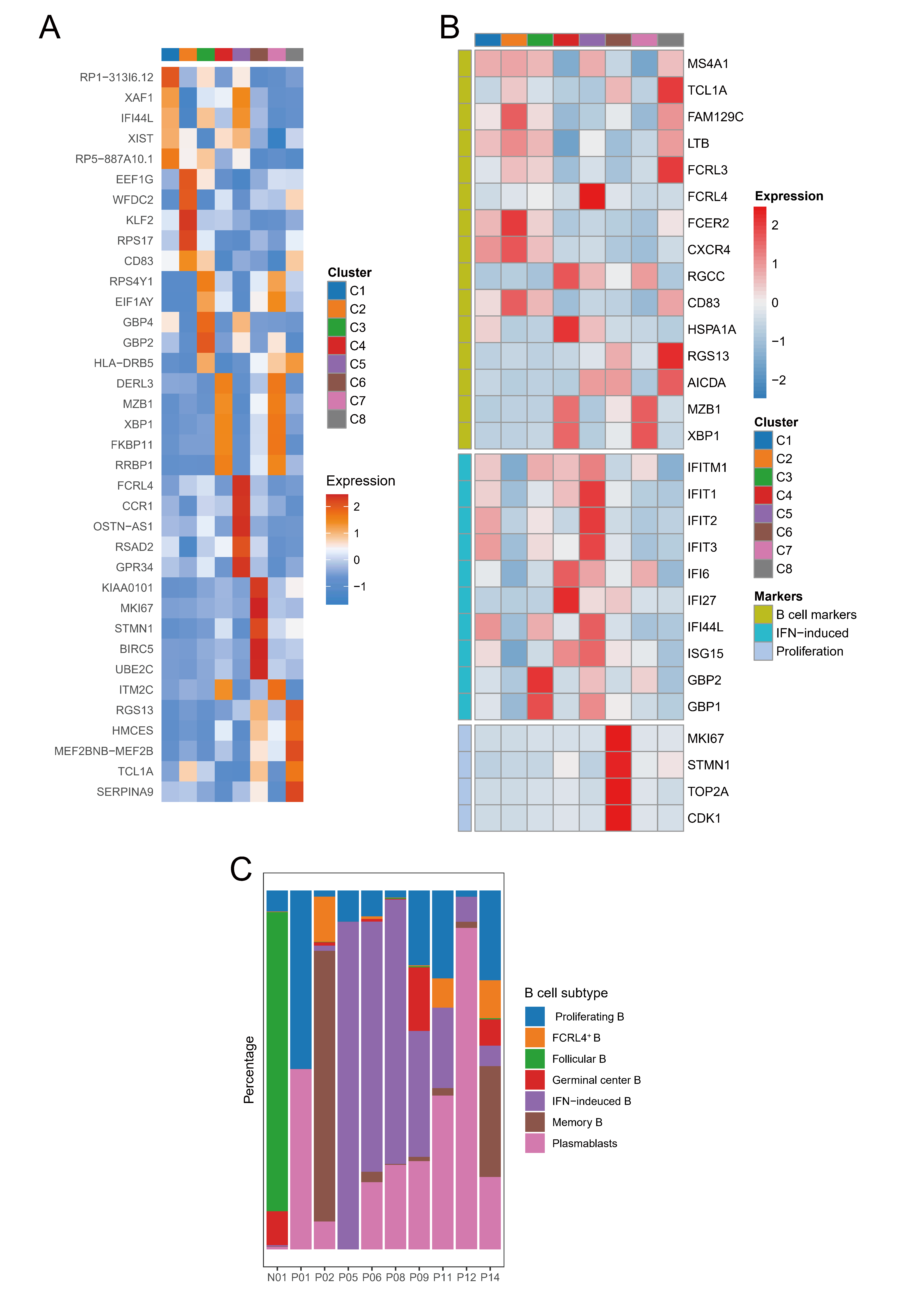


**Fig. S4** (A) Heatmap shows the top 5 differently expressed genes of each of eight cluster of B cells. (B) Heatmap shows the expression levels of conserved gene markers or functional gene markers in eight B cell subtypes. (C) The proportion of seven B cell subtypes in different sample origins.


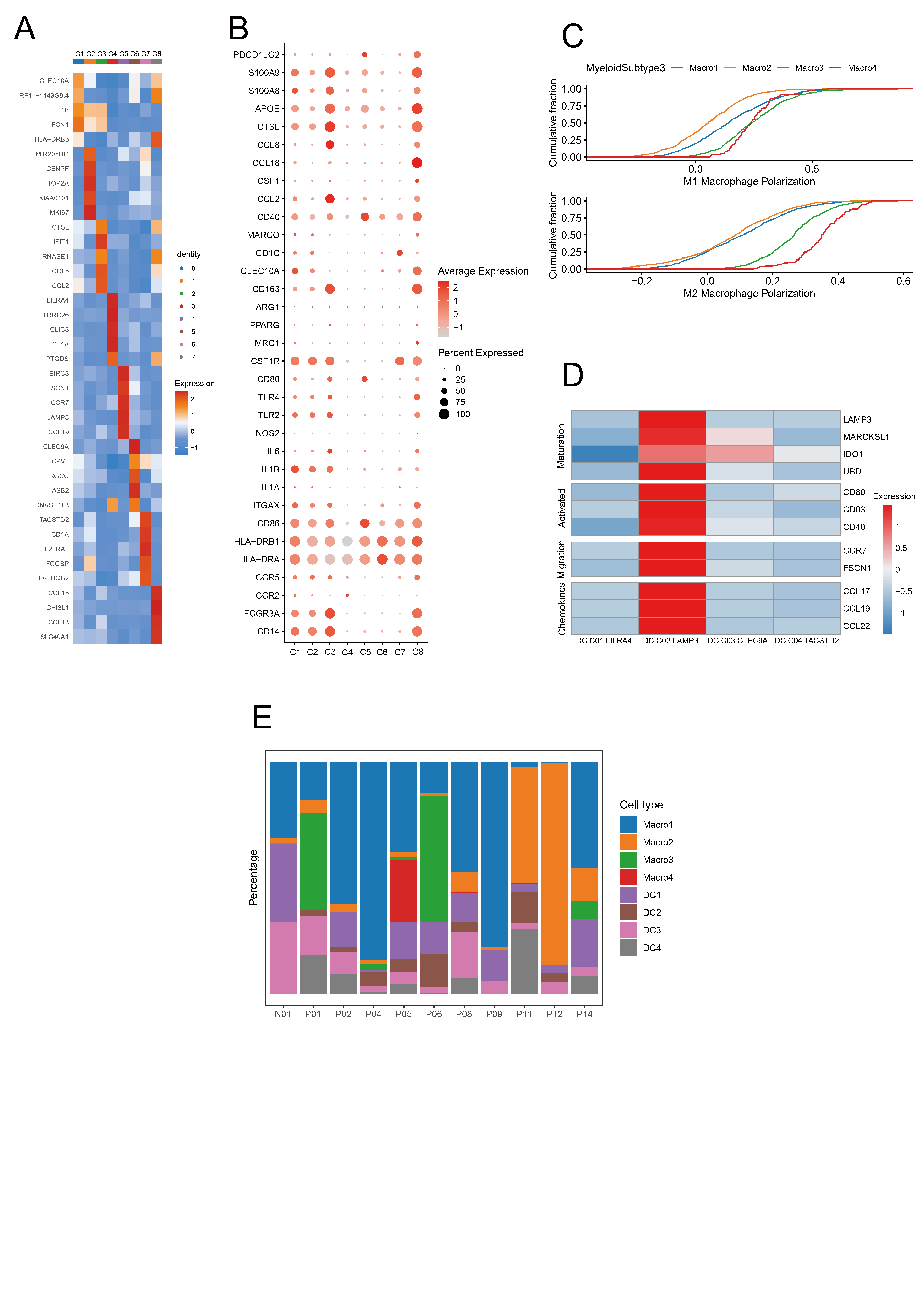


**Fig. S5** (A) Heatmap shows the top 5 differently expressed genes of each of eight cluster of myeloid cells. (B) Dot plot shows the expression levels of selected gene markers in eight myeloid cell subtypes. (C) Cumulative distribution function shows the distribution of M1 (upper panel) and M2 (lower panel) polarization scores in four macrophages subsets. A rightward shift of the curve indicates increased M1 or M2 polarization scores. (D) Heatmap shows the the expression levels of chemokines, migration, activation, and maturation gene signatures in four dendritic cell subsets. (E) The proportion of eight myeloid cell subtypes in different sample origins.

| **Table S1.** scRNA-Seq parameters of study samples | | | | | |
| --- | --- | --- | --- | --- | --- |
| **ID** | **Histology** | **10X Version** | **No. Of UMI** | **No. of cell** | **UMI per cell** |
| N01 | Normal tissue | 2.0 | 23341939 | 2583 | 9037 |
| P01 | Tumor | 2.0 | 112899534 | 2217 | 50924 |
| P02 | Tumor | 2.0 | 46012984 | 6092 | 7553 |
| P04 | Tumor | 2.0 | 8930200 | 320 | 27907 |
| P05 | Tumor | 2.0 | 24831459 | 1842 | 13481 |
| P06 | Tumor | 2.0 | 22665938 | 2222 | 10201 |
| P08 | Tumor | 2.0 | 24955311 | 5313 | 4697 |
| P09 | Tumor | 2.0 | 12251927 | 834 | 14691 |
| P11 | Tumor | 2.0 | 40914978 | 2589 | 15803 |
| P12 | Tumor | 2.0 | 28407803 | 1914 | 14842 |
| P14 | Tumor | 2.0 | 32323223 | 2497 | 12945 |
| Note：UMI, unique molecular identifier. | | | | | |

| **Table S2.** Patient characteristics of the samples included in this study | | | | | | |
| --- | --- | --- | --- | --- | --- | --- |
| **ID** | **Histology** | **TNM**  **classifications**^*^ | **Clinical stage**^*^ | **Smoking status** | **Pretreatment**  **EBV DNA**  **（copies/mL）** | **EBERs status** |
| N01 | CNP | T3N1M0 | - | No | 0 | - |
| P01 | NKNPC | T2N3M0 | IVA | Yes | 1.16*10^4^ | + |
| P02 | NKNPC | T3N1M0 | III | No | 1.91*10^3^ | + |
| P04 | NKNPC | T4N3M1 | IVB | Yes | 0 | + |
| P05 | NKNPC | T3N0M0 | III | No | 1.38*10^3^ | + |
| P06 | NKNPC | T2N2M0 | III | No | 1.23*10^4^ | + |
| P08 | NKNPC | T2N1M0 | II | Yes | 2.84*10^3^ | + |
| P09 | NKNPC | T2N3M0 | IVA | No | 0 | + |
| P11 | NKNPC | T1N1M0 | II | Yes | 0 | + |
| P12 | NKNPC | T4N2M0 | IVA | No | 9.90*10^3^ | + |
| P14 | NKNPC | T4N2M1 | IVB | No | 7.00*10^6^ | + |
| ^*^Patients were staged according to the 8^th^ AJCC staging manual.  Abbreviation: CNP = chronic nasopharyngitis; NKNPC = non-keratinizing nasopharyngeal carcinoma. | | | | | | |

| **Table S3.** Summary of gene signature datasets | |
| --- | --- |
| **Cancer hallmark gene sets** | http://www.gsea-msigdb.org/gsea/msigdb/collections.jsp |
| **CD8+ T cell gene signatures** |  |
| Naive signature | CCR7, TCF7, LEF1, SELL |
| Cytotoxic signature | PRF1, GZMB, GZMA, IFNG, NKG7, GNLY |
| Effe-Memory signature | CD27, CD28, CCR7, CCR5, SELL, FAS |
| Exhaustion signature | HAVCR2, LAG3, TIGIT, CTLA4, PDCD1, ENTPD1, TOX |
| IFN-γ signature | IDO1, CXCL10, CXCL9, HLA-DRA, STAT1, IFNG |
| **CD4+ T cell gene signatures** |  |
| IL2R | IL2RA, IL2RB, IL2RG |
| Inhibitory | EBI3, HAVCR2, LAG3, TIGIT, CTLA4, ENTPD1, LAYN |
| Co-stimulatory | ICOS, TNFRSF4, TNFRSF18, TNFRSF9 |
| **PanCancer Immune Profiling** | https://nanostring.com/products/ncounter-assays-panels/oncology/pancancer-immune-profiling/ |
| **M1 and M2 Signatures** |  |
| M1 signatures | IL12A, IL23A, IL12B, TNF, IL6, CD86, HLA-DRA, IL1B, MARCO, NOS2, IL12RB1, FCGR1A, CD80, IL23A, CXCL9, CXCL10, CXCL11, CD86, IL1A, IL1B, IL6, TNF, HLA-DRB1, CCL5, IRF5, IRF1, CD40, IDO1, KYNU, CCR7, PTPRC, CD68, CSF1R, HLA-DRB5, LY75, CD14 |
| M2 signatures | ARG1, ARG2, IL10, FCGR2B, CD163, FCGR2A, CD200R1, PDCD1LG2, CD274, MARCO, CSF1R, MRC1, IL1RN,  IL1R2, IL4R, CCL4, CCL13, CCL20, CCL17, CCL18, CCL22, CCL24, LYVE1, VEGFA, VEGFB, VEGFC, FIGF, EGF, CTSA, CTSB, CST3, CTSD, TGFB1, TGFB2, TGFB3, MMP14, MMP19, MMP9, CLEC7A, WNT7B, FASLG, TNFSF12, TNFSF8, CD276, VTCN1, MSR1, FN1, IRF4, PTPRC, CD68, CSF1R, HLA-DRA, LY75, CD14 |
| **Dendritic cell signatures** |  |
| Chemokines | CCL17, CCL19, CCL22 |
| Migration | CCR7, FSCN1 |
| Activation | CD80, CD83, CD40 |
| Maturation | LAMP3, MARCKSL1, IDO1, UBD |
| **Myeloid inte immunity** | https://www.nanostring.com/products/gene-expression-panels/gene-expression-panels-overview/ncounter-myeloid-innate-immunity-panel |
| **Interferon response** |  |
| Type I Interferon response | IRF1, IFIH1, IFITM3, DDX58, IFI44L, IFI6, IFITM2, NAMPT, OASL, RTP4, TREX1, ADAR, FAM46C, LY6E, MCOLN2, APOBEC3G, IL15, ISG15, MX1, TLR3 |
| Type II Interferon Response | IFNG, CXCL9, CXCL10, STAT1, HLA-A, HLA-B, PDCD1, CDKN1A, MYC, SMAD7, IRF1 |
